# Multiple genome analyses reveal key genes in Vitamin C and Vitamin D synthesis and transport pathways are shared

**DOI:** 10.1038/s41598-019-53074-9

**Published:** 2019-11-14

**Authors:** Wei Dong, Cheng Tian, Yan Jiao, Savannah Blackwell, Ge Lou, Arnold Postlethwaite, Weikuan Gu, Dianjun Sun

**Affiliations:** 1 Department of Orthopaedic Surgery and Biomedical Engineering, University of Tennessee Health Science Center (UTHSC), 956 Court Av, Memphis, TN 38163 USA; 2 Department of Gynecology, Harbin Medical University Cancer Hospital, Haping Road, Harbin, Heilongjiang 150081 China; 3 Department of Medicine, University of Tennessee Health Science Center (UTHSC), 956 Court Av, Memphis, TN 38163 USA; 4 Research Service, Veterans Affairs Medical Center, 1030 Jefferson Avenue, Memphis, TN 38104 USA; 5 Center for Endemic Disease Control, Chinese Center for Disease Control and Prevention, Harbin Medical University; Key Laboratory of Etiologic Epidemiology, Education Bureau of Heilongjiang Province & Ministry of Health (23618104), 157 Baojian Road, Harbin, Heilongjiang 150081 China

**Keywords:** Metabolic pathways, Molecular medicine

## Abstract

Vitamin C (VC) and vitamin D (VD) have been widely used as the dietary supplements and in treatment of diseases both independently and in combination. Whether there is a connection between their pathways is critical for their therapeutic applications. Using whole-genome expression profiles, we performed multiple measures of associations, networks, eQTL mappings and expressions of key genes of interest in VC and VD functions. Several key genes in their pathways were found to be associated. *Gc* and *Rgn* play important roles connecting VC and VD pathways in mice. The r values of expression levels between *Gc* and *Rgn* in mouse spleen, liver, lung, and kidney are 0.937, 0.558, 0.901, and 0.617, respectively. The expression QTLs of *Gc* and *Rgn* are mapped onto the same locations, i.e., 68–76 MB in chromosome 7 and 26–36 MB in chromosome 9. In humans, there are positive correlations between *CYP27B1* and *SLC23A1* expression levels in kidney (r = 0.733) and spleen (r = 0.424). *SLC23A2* and *RXRA* are minimally associated in both mouse and human. These data indicate that pathways of VC and VD are not independent but affect each other, and this effect is different between mice and humans during VC and VD synthesis and transportation.

## Introduction

Vitamin C (VC) and vitamin D (VD) are indispensable for optimal health in humans. More than half a century ago, researchers found that VD deficiency could lead to rickets, and VC deficiency could lead to scurvy^1,2^. More recently many researchers have focused on the functions of VD and VC in different diseases and/or in different tissues^3–7^. Converging evidence suggests that VC and VD may exert similar effects in some circumstances. Wei *et al*. reported that there may be a moderate inverse association between dietary VC intake and non-alcoholic fatty liver disease (NAFLD) in middle-aged and older adults^3^. A different group found that timely initiation of VD supplementation is a critical determinant of treatment outcome in NAFLD^4^. VC and VD are each important in modulating bronchial asthma^5^. In addition, VD and VC both have pleiotropic effects on the immune, cardiovascular, and neurological systems and also share antineoplastic activity^6,7^. In spite of similarities between VD and VC functions in some diseases and human health, their applications in therapeutics and diet supplementation have largely been assessed independently, with little attention devoted to their potential interaction.

### Key genes in VD pathways

With the help of ultraviolet B (UVB), both mouse and human body can synthesize Vitamin D3 from dehydrocholesterol (DHC). 7-dehydrocholesterol reductase (*Dhcr7*) is one of the regulatory genes in DHC production. Humans also obtain Vitamin D3 through diet. In the body, vitamin D3 is first converted to 25-hydroxyvitamin D (25(OH)D) and then to 1α,25-dihydroxyvitamin D(1,25(OH)D3)^8^. *Gc* is the key gene to producing VD binding protein (DBP), which binds 25(OH)D and 1,25(OH)D3 in plasma. Vitamin D receptor (*Vdr*) in target cells binds specifically to 1,25(OH)D3 with high affinity^9^. A large number of genes are involved in VD molecular pathways in target cells. The most important ones are *Cyp24a1*, *Trpv6*, *Cyp27b1*, *Cyp2r1*, and *Rxra* which are studied in our lab^8–10^. Among them, Rxra is an obligatory co-receptor for *Vdr* mediating transcription^11^.

### Key genes in VC synthesis and transport

Mice can synthesize vitamin C *de novo*. The pathway to synthesize vitamin C is through D-glucuronate, in which L-gulonate and L-gulono-γ-lactone serve as intermediate metabolites. Humans, some primates, and guinea pigs cannot synthesize vitamin C because their *Gulo* gene, which produces L-*gulo*no-γ-lactone oxidase, is mutated. Senescence-marker protein-30 (SMP30) is essential for the conversion of L-*gulo*nate to L-*gulo*no-γ-lactone. SMP30 is produced by the regucalcin (*Rgn*) gene^12^. Two sodium-dependent transporters *Slc23a1* and *Slc23a2* (also referred to as Svct1 and Svct2), are important regulators in VC transport as well^13^. When *Rgn* or *Slc23a2* are knocked out, mice cannot synthesize VC^14^. In this research we chose to study the role of *Gulo*, *Rgn*, *slc23a1*, and *Slc23a2* in VC function and the role of *Vdr*, *Gc*, *Cyp24a1*, *Trpv6*, *Cyp27b1*, *Cyp2r1*, *Dhcr7*, and *Rxra* in VD function.

### History of BXD strains

For over half a century, recombinant inbred (RI) strains of mice have been well-studied as models in biomedical research. In this research, we chose BXD RI strains, which represent the best developed animal model and have made significant contributions to the study of human diseases. The BXD families of RI strains were derived by crossing C57BL/6 J (B6) and DBA/2 J (D2) mice. Multiple generations of individuals from the F2 group were produced^15^. Theoretically, after more than 20 generations of inbreeding, most genetic sites of inbred animals should be homozygous. The BXD database can be downloaded from GeneNetwork, a public data source website that is a large whole genome expression resource platform. Using GeneNetwork, we worked to find potential overlap in synthesis and transport pathways of VC and VD.

## Results

### The crosstalk of key genes in gene network between VC and VD pathways

From the GeneNetwork platform, we first obtained the expression levels of VC key genes (*Gulo*, *Rgn*, *Slc23a1*, and *Slc23a2*) and VD key genes (*Vdr*, *Gc*, *Cyp24a1*, *Trpv6*, *Cyp27b1*, *Cyp2r1*, *Dhcr7*, and *Rxra*) from whole genome mRNA expression profiles in four different mouse and human organ tissues (spleen, liver, lung and kidney). Next, network graphs of these key genes were created using a threshold of absolute values greater than 0.35 as we previously reported^16^.

**Figure 1:**
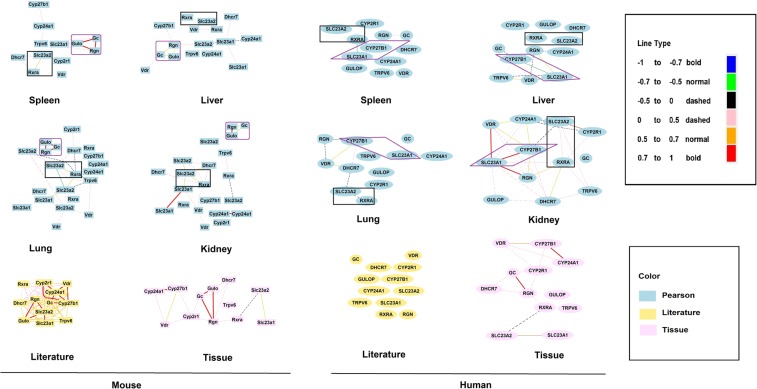
Different correlation levels among VC and VD key genes. The left portion shows correlations in mice and the right portion shows correlations in humans. Graphs in blue are Pearson’s correlations in four types of tissues. Literature correlation is presented in yellow and tissue correlation in pink. Different colors and thickness of line show different levels of correlation.

As shown in Fig. 1, in the pathways for VC and VD synthesis, it is obvious that *Gc*, *Rgn*, and *Gulo* in mice are tightly connected together compared to other genes in Pearson’s r value in the four tissues, especially in spleen and lung. The correlation between *Gc*, *Rgn,* and *Gulo* are all connected in red lines, depicting the positive correlations among them. We also examined correlations among these key genes reported in the literature for mouse and human tissue (Fig. 1). Correlations among these genes were more frequently reported for mice Correlations in mouse tissue showed similarity of expression in the same tissues among these key genes. GeneNetwork and the literature both support positive correlations among *Gc*, *Rgn* and *Gulo*, raising the question as to whether *Gc* is the key gene that overlaps in the VC and VD pathways in mice. However, since humans cannot synthesize VC due to a mutation in the *Gulo* gene, such a correlation is not present in humans. Furthermore, *GC* and *RGN* are not connected in humans.

For the genes involved in transport of VC and VD, it seems that *SLC23A1* is connected to *CYP27B1* in humans in the spleen, liver, and kidney. The figures all show strong correlations between *SLC23A1* and *CYP27B1* in the tissues, except for the lung. The correlation is especially strong in the kidney. However, such a strong connection is lacking in mice. However, a clear connection between *Slc23a2* and *Rxra* is present in both the mouse and human version of the genes (*SLC23A2* and *RXRA*) are also connected. Thus, in post-synthesizing pathways there are both similarities and differences between humans and mice. Of note, in the human literature and tissue correlation analyses, connections between *SLC23A1* and *CYP27B1* are lacking.

### Differences and similarities in patterns of correlation among key VC and VD genes

Since initial analysis of correlations among key genes in the VD and VC pathways showed both similarities and differences between mice and humans, we further examined in detail the correlation matrix among these genes in mouse spleen (N = 81), liver (N = 32), lung (N = 49), and kidney (N = 55) using the GeneNetwork platform with the Pearson coefficient^17^.

**Figure 2:**
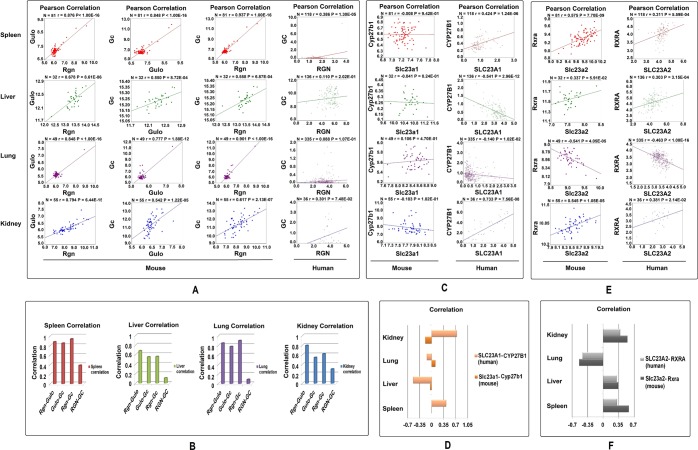
The correlations between paired key genes in four tissues, the spleen, liver, lung, and kidney. The number of samples, R value, and P value of each group are shown at the top of each graph. (**A**) Pearson’s correlations between Gulo and Rgn in mice, Gc and Gulo in mice, Gc and Rgn in mice, and GC and RGN in humans. (**B**) Correlation values between paired genes in four tissues. (**C**) Pearson’s correlations between SLC23A1 and CYP27B1 in humans, and Slc23a1 and Cyp27b1 in mice in the four tissues. (**D**) Correlation values of paired genes. (**E**) Pearson’s correlations between Rxra and Slc23a2 in mice and RXRA and SLC23A2 in humans. (**F**) Correlation values of paired genes in our tissues.

Figure 2A shows correlations in different tissues between *Gulo* and *Rgn*, *Gc* and *Gulo*, and *Gc* and *Rgn* in mouse and *GC* and *RGN* in human. Figure 2B summarizes the correlation between the expression levels of these genes. Correlations (r values) between *Gulo* and *Rgn* in mouse spleen, liver, lung, and kidney were 0.876, 0.678, 0.848, and 0.794, respectively. Correlations between *Gc* and *Gulo* in these four tissues in mice were 0.848, 0.550, 0.777, and 0.542, respectively. The R values between *Gc* and *Rgn* in these tissues of mice are 0.937, 0.558, 0.901, and 0.617, respectively. All of these correlations are statistically significant at less than 0.001. *Rgn*, *Gc*, and *Gulo* are highly expressed in mouse liver, and their correlations are more than 0.5 in this tissue. These detailed analyses in the four tissues confirm the positive correlation among these three genes. At the same time, the correlations of *GC* and *RGN* in humans are 0.386, 0.110, 0.088, and 0.301 (Fig. 2B), which means *GC* and *RGN* have a low degree of linkage in humans (as expected, since humans cannot synthesize VC). Further, we have found a similarly strong correlation between Gc and Rgn in rat tissues (Supplementary Fig. S1).

Figure 2C shows the detailed correlation between *Cyp27b1* and *Slc23a1* in mice and *CYP27B1* and *SLC23A1* in humans. Figure 2D summarizes correlations among these gene pairs in the four tissues. In humans, there are obvious positive correlations between the expression levels of *CYP27B1* and *SLC23A1* in kidney and spleen, with r values of 0.733 and 0.424. However, in liver the relation between these two genes is negative. In mice, there is no correlation between *Cyp27b1* and *Slc23a1*, with r values between −0.183 and 0.106. Either positive or negative, at these low levels of R values, it means that they do not correlate. In liver the number of shared probes is the least between *Gc* and *Rgn*. However, even such a small number of shared genes are difficult to find when the two genes are not related. We have shown in the supplementary data that in pairs of non-relevant genes, there are no shared probes that are closely related to their expression levels.

Figure 2E,F show the correlation of expression levels between *Rxra* and *Slc23a2* in mice and *RXRA* and *SLC23A2* in humans. These weak connections are shown in a graphic gene network between the PAK pathway and RXRA in four tissues (Supplementary Fig. S2). In kidney, liver, and spleen, these gene pairs show positive correlations at the expression level while in lung they have a negative correlation. The P values of correlations in these tissues between humans and mice are similar, with a P value of 0.2890.

Data shown in Figs 1 and 2 suggest there are both similarities and differences in regulation of synthesis and transport pathway between VD and VC. In animals where *Gulo* is functional, *Rgn* and *Gc* have some linkage in VC and VD pathways. The correlation between *Rgn* and *Gc* in humans in these tissues is significantly lower than that in mice. The P value of correlations between mice and humans is 0.0173. However, due to the non-functional *GULO* gene in human, almost all the VC is obtained from the diet, thus *RGN* and *GULO* do not play any role in linkage of the VC and VD synthetic pathways. After VC intake, *SLC23A1* and *SLC23A2* start to transport VC to different tissues, and then the main linkage of VC and VD is between *Rxra* and *Slc23a2* in mice and *RXRA* and *SLC23A2* in humans. In addition, there is an alternative pathway that may connect VC and VD through *SLC23A1* and *CYP27B1*. Such a linkage has not been demonstrated in mice.

### Significant variations in the similarities of top 100 probes that are most correlated to key genes in the VC and VD pathways in mice

**Figure 3:**
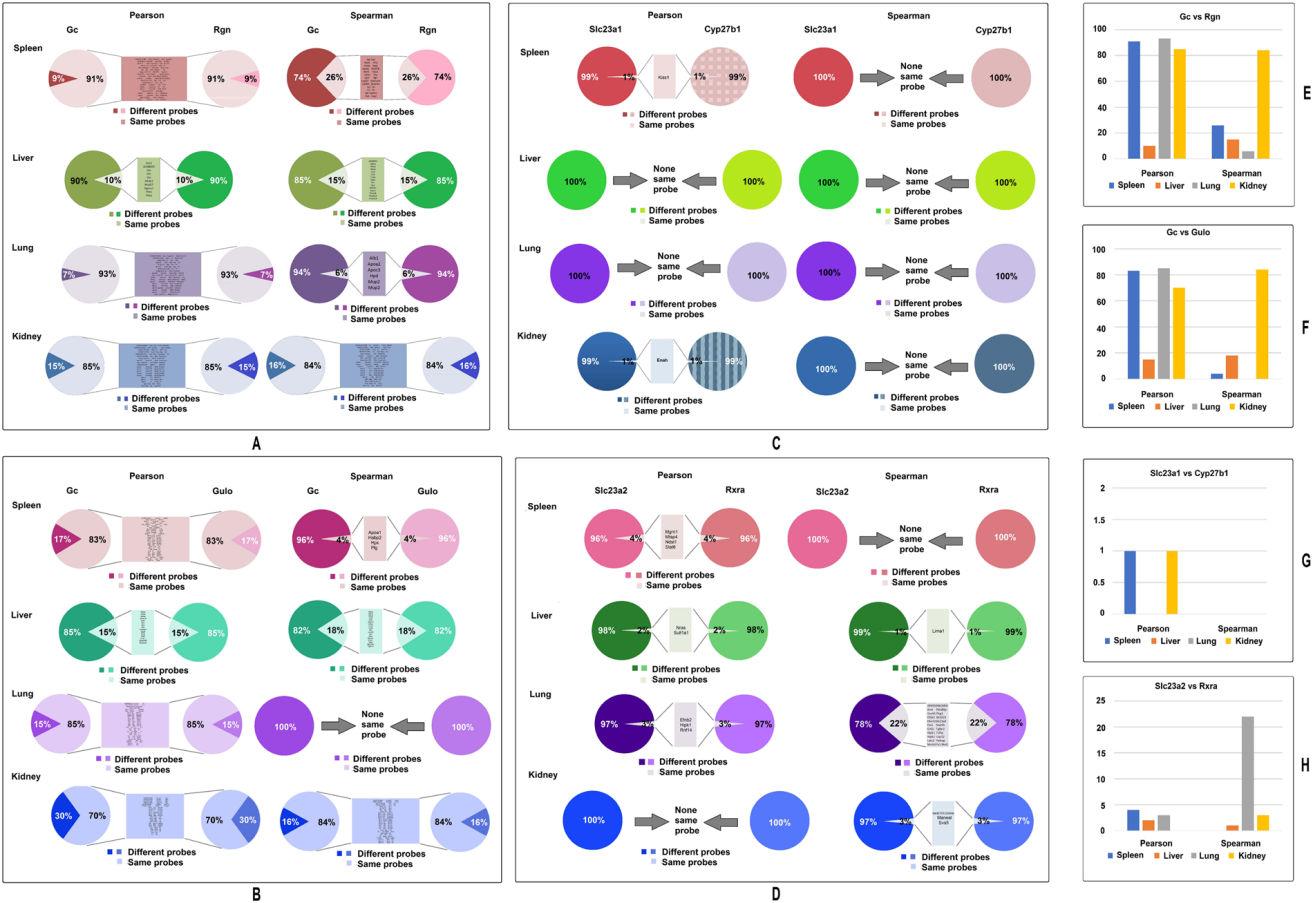
The similarities between *Gc* and *Rgn*’s top 100 probes in spleen, liver, lung, and kidney of mice. Each group shows the percentage of same and different probes in the top 100 probes, and the names of probes which are the same between a pair of genes.

The linkage between key genes in VD and VC pathways and significant differences in their patterns of correlations raises a question as to what the similarities and differences among the top genes are and which expression levels are closely associated with the key genes. The method of exploration was to compare the top 100 most correlated genes between each correlated pair of genes, as we previously explained except we obtained the top 100 instead of 50^16^. Again, using GeneNetwork tools, the top 100 probes that have their expression levels most correlated with that of the expression level of each gene were identified. We first compared the similarities between the top 100 probes from *Gc* and *Rgn*. For the correlation, we used both Pearson and Spearman’s correlation (Fig. 3A,E). We found that in the kidney more than 80 probes of *Gc* and *Rgn* are the same using both Pearson’s and Spearman’s correlation coefficients. In the spleen the Pearson correlation showed that as many as 91 probes are the same. This extremely similar phenomenon is usually rare when other genes are compared to each other. The results suggest that *Gc* and *Rgn* have a strong relationship in these tissues. In liver the number of shared probes is the least between Gc and Rgn. However, even such a small number of shared genes are difficult to find when the two genes are not related. We have shown in the supplementary data that in the pairs of non-relevant genes, there are no shared probes that are closely related to their expression levels (Supplementary Table S2).

Similarly, large numbers of the same genes were found among the top 100 most correlated probes to *GC* and *Gulo* among four tissues (Fig. 3B,F). Similarly, large numbers of the same genes were found among the top 100 most correlated probes to GC and Gulo among three tissues except in liver. By Pearson coefficient, the number of same genes is 83, 15, 85, and 70 in spleen, liver, lung, and kidney, respectively. By Spearman’s coefficient, the numbers were 4, 18, 0, and 84, respectively. In order to re-affirm the relationship in the liver, we have done the same analysis with data from rat liver that confirmed the finding from mouse liver (Supplementary Fig. S3).

The top 100 most correlated genes to *Slc23a1* and *Cyp27b1* are compared in mice (Fig. 3C,G). While one gene in mouse spleen and kidney were found to be the same between *Slc23a1* and *Cyp27b1* by Pearson coefficient, the same result was not found using the Spearman’s coefficient.

Genes from the top 100 most correlated genes were found to be the same between *Rxra* and *Slc23a2* in mice using the two statistical coefficient methods (Fig. 3D,H). In mice the number of the same genes found by using Pearson’s coefficient were 4, 2, 3, and 0 in spleen, liver, lung, and kidney respectively. Using Spearman’s coefficient, the same genes were 0, 1, 22, and 3, respectively. In contrast, there were no same probes among the top 100 genes derived from genes in unrelated pathways, such as the top 100 genes from Il1rn, Avpr1a, Vdr, and Lep (Supplementary Table S2).

### Significant variations in the similarities of top 100 probes that are most correlated to key genes in the VC and VD pathways in humans

**Figure 4:**
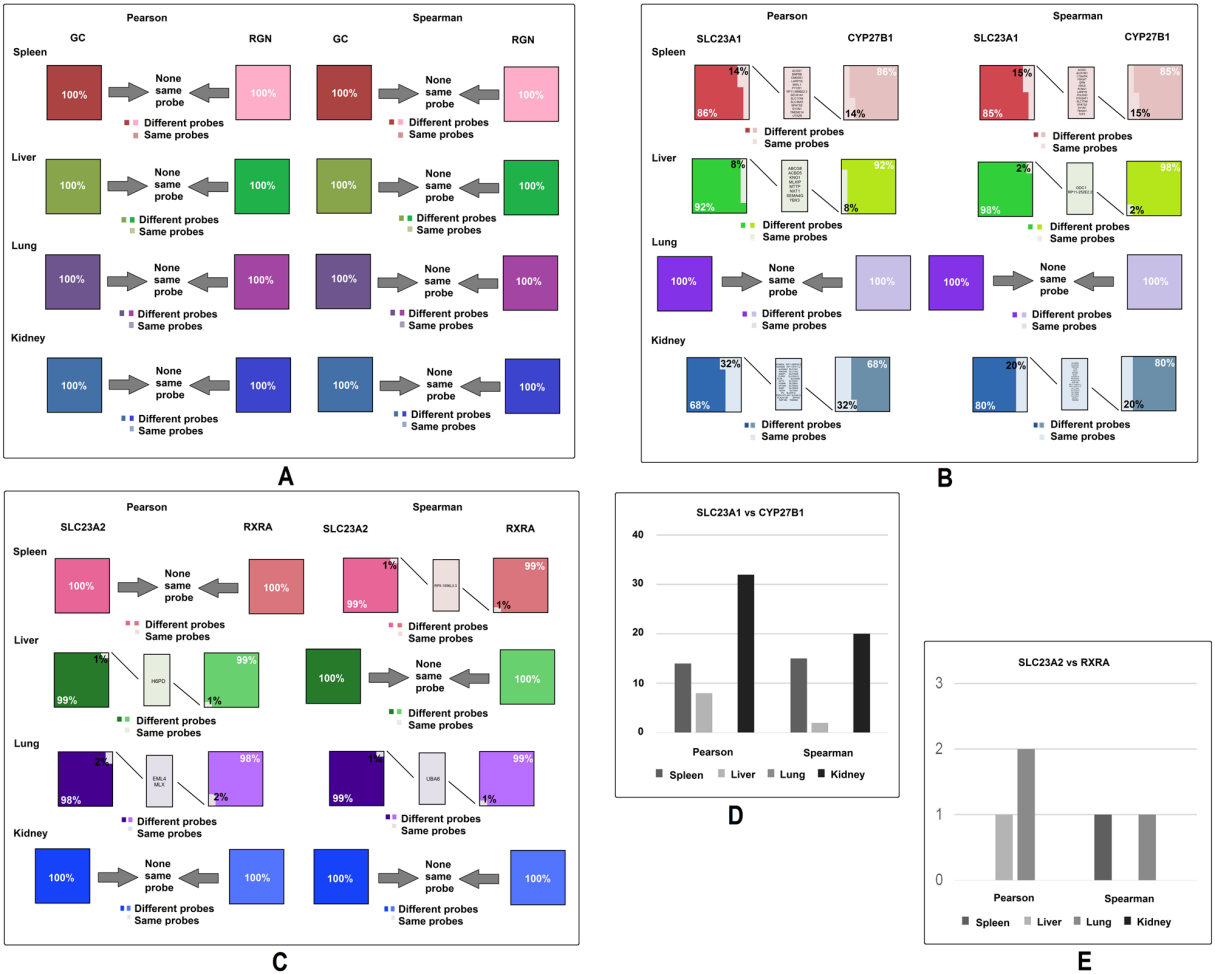
The similarities between *Gc* and *Rgn*’s top 100 probes in spleen, liver, lung, and kidney of humans. Each group shows the percentage of same and different probes in the top 100 probes, and names of the probes which are the same between genes in the pair. (**A**) Comparison of top 100 most closely correlated genes between *GC* and *RGN* in the four tissues, using both Pearson’s and Spearman’s coefficient. (**B**) Comparison of top 100 most closely correlated genes between *SLC23A1* and *CYP27B1* in the four tissues, using both Pearson’s and Spearman’s coefficient. (**C**) Comparison of top 100 most closely correlated genes between *SLC23A2* and *RXRA* in four tissues, using both Pearson’s and Spearman’s coefficient. (**D**) Number of the same genes among top 100 most closely correlated genes between *SLC23A1* and *CYP27B1* in four tissues, using both Pearson’s and Spearman’s coefficient. (**E**) Number of the same genes among top 100 most closely correlated genes between *SLC23A2* and *RXRA* in four tissues, using both Pearson’s and Spearman’s coefficient.

Using the same method from the mouse analysis, we analyzed the top human probes of three gene pairs, *GC* and *RGN*, *SLC23A1* and *CYP27B1*, and *RXRA* and *SLC23A2*. Surprisingly, there is no identical probe in the top 100 most correlated probes of *GC* and *RGN*, regardless of whether Pearson or Spearman’s calculations are employed (Fig. 4A). These data support the conclusion that the correlation in mice between this pair of genes does not exist in humans. P values comparing the number of genes in mice (Fig. 3A,E) and in humans by Pearson’s coefficient was 0.0398 and Spearman’s was 0.1591; the combination of the two is 0.0085.

Next, we compared the top 100 most correlated genes to *SLC23A1* and *CYP27B1* in humans (Fig. 4B,D). A large number of probes were found to be the same between *SLC23A1* and *CYP27B1* among the spleen, liver, and kidney using both Pearson’s and Spearman’s correlations coefficient, except the lung. The number of the same genes in spleen, liver, lung, and kidney by Pearson’s coefficient were 14, 8, 0 and 32, respectively, and by Spearman’s coefficient were 15, 2, 0, and 20, respectively. P values comparing the number of genes in mice (Fig. 3C,G) and in humans by Pearson’s is 0.1422, by Spearman’s is 0.1548, and the combination of the two is 0.0235. These data support the conclusion that the role between *SLC23A1* and *CYP27B1* in linkage between VD and VC pathway is more important in humans than in mice.

Lastly, we compared the top 100 most correlated genes to *RXRA* vs *SLC23A2* in humans (Fig. 4C,E). While in humans there are some genes that are the same between the top 100 most closely correlated genes of one to the other, there are fewer numbers of identical genes in humans than in mice. The number of identical genes in spleen, liver, lung, and kidney by Pearson’s coefficient were 0, 1, 2 and 0, respectively, and by Spearman’s coefficient were 1, 0, 1, and 0, respectively. P values comparing the number of genes in mice and in humans by Pearson’s coefficient were 0.1817, by Spearman’s is 0.3216, and the combination is 0.1813. Thus, a slightly weaker correlation between this pair of gene exists in humans and a stronger one in mice (Fig. 3D,H).

### eQTL mapping in *Gc* and *Rgn* in mouse

**Figure 5:**
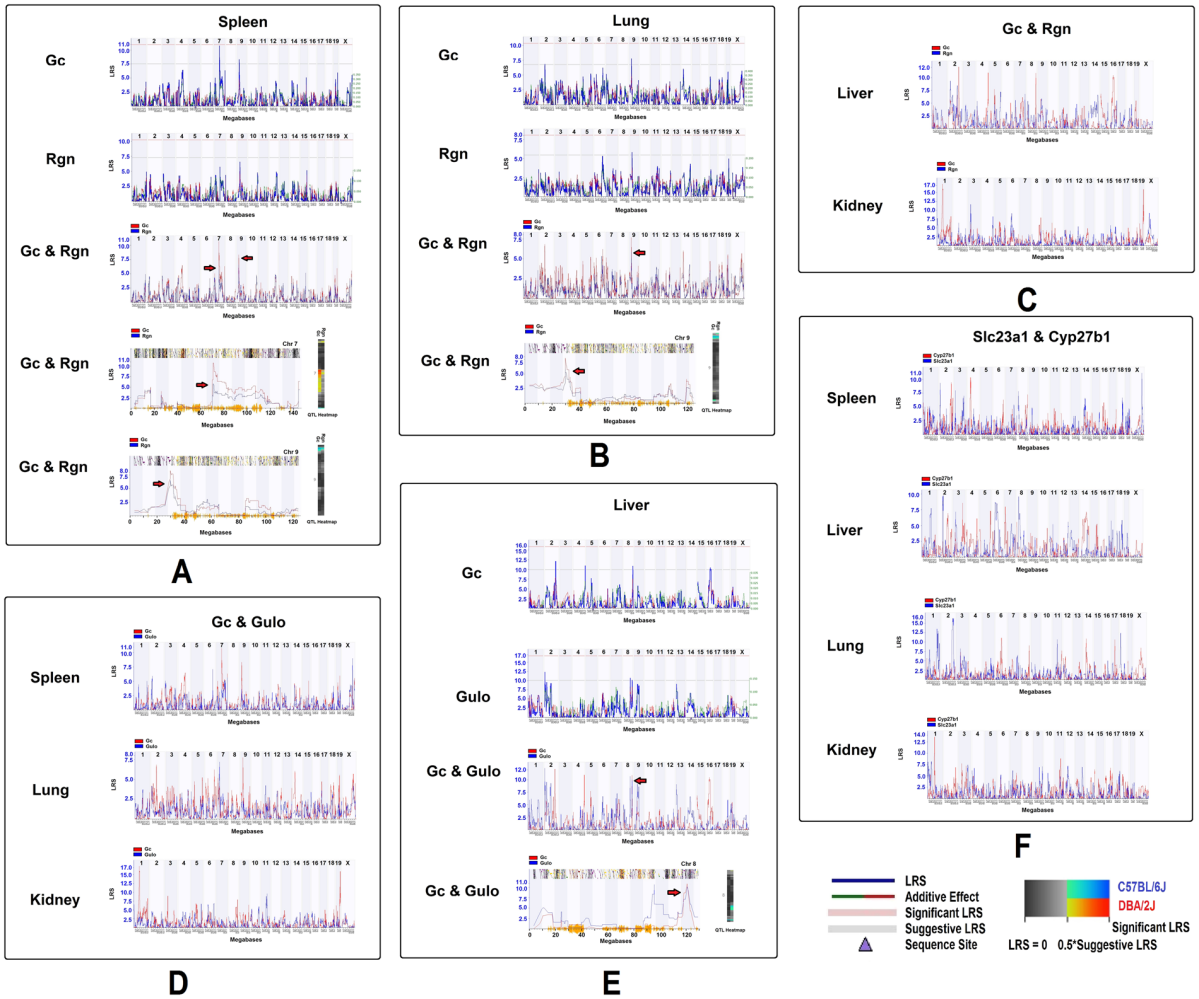
Locations of eQTLs that regulate the expression levels of key genes in mice. On the left of each picture is the LRS, which measures the association of linkage between the expression levels of key genes and particular genotype markers on mouse chromosomes. Each heatmap is shown at the right of the eQTL figure. (**A**) The locations of eQTLs on chromosome 7 and 9 that regulate the expression levels of *Gc* and *Rgn* in spleen. (**B**) Locations of eQTLs that regulate the expression levels of *Gc* and *Rgn* on chromosome 9 in lung of mouse. (**C**) eQTL mapping of expression levels of *Gc* and *Rgn* in liver and kidney. (**D**) The potential eQTL that regulate *Gc* and *Gulo* in tissues of spleen, lung and kidney. (**E**) The eQTL locations that regulate the expression of *Gc* and *Gulo* on chromosome 8 in liver. (**F**) The eQTL mapping of *Slc23a1* and *Cyp27b1* in four tissues.

Due to the availability of genotypes of BXD strains, we were able to examine the regulatory loci of these key genes in mice. The eQTL of *Gc* and *Rgn* from the four tissues studied were obtained from GeneNetwork using an interval mapping tool with 1000 permutations and with all the expression data from BXD strains without removing outliers from data from the four tissues. We conducted two separate mapping procedures, mapping *Gc* and *Rgn* individually and mapping them together. When mapped individually, the result suggested that *Gc* has a possible eQTL in both chromosome 7 and chromosome 9 in spleen (Fig. 5A). The highest LRS (Likelihood ratio statistic) of *Rgn* is also in the same position on chromosomes 7 and 9 although the eQTL did not reach a significant LRS. When mapping *Gc* and *Rgn* together, the result showed they are in the same location, 68–76 MB in chromosome 7 and 26–36 MB in chromosome 9 (Fig. 5A, picture at bottom row). While in the lung, eQTL from *Gc* and *Rgn* both reached suggestive levels in chromosome 9, in a similar location as that in the spleen, between 28–34 MB (Fig. 5B). The heatmap showed the same results as that of the eQTL mapping (Fig. 5A,B). No significant eQTL was identified from liver or kidney (Fig. 5C). Although the eQTL of *Gc* and *Gulo* from spleen, lung, and kidney rose to a significant level, they were mapped on chromosome 9 in both the spleen and lung (Fig. 5D). In addition, in liver, an eQTL on chromosome 8 was obtained by individual and combined mapping (Fig. 5E). No significant eQTL was identified between *Slc23a1* and *Cyp27b1*, although a potential locus on chromosome 8 was mapped from both spleen and liver (Fig. 5F).This analysis identified a strong association between *Gc* and *Rgn*, *Gc* and *Gulo*, and between *Slc23a1* and *Cyp27b1*. This result further enhances the assumption that there are connections between VD and VC pathways in mice.

### Key genes were expressed differently in one pathway when the key gene in another pathway was affected

The expression levels of VC and VD key genes in Sfx mice and Balb/c mice were examined to determine whether there is a difference in expression levels in VD key genes when a VC key gene was knocked out. As is shown in Table 1, all the groups showed that when Gulo was knocked out, no matter whether the supplied drinking water contained VC or not, some VD key genes were expressed differently in the Sfx group than the Balb/c group. The two analysis in femurs showed similar differences, while the differences between two femur groups are the mice age and the analyzed methods.

**Table 1:** Different gene expression levels of VC and VD key genes in Sfx with /without VC treatment and Balb/c mice.

Tissue	Symbol	Sfx with VC		Sfx without VC		Balb/c		F Log Ratio	M Log Ratio
		F	M	F	M	F	M		
Femur (7 weeks mice)	Vdr	809 ± 87.7	780 ± 73	810.7 ± 102.8	743 ± 185	623.1 ± 13.4	557.6 ± 50	—	—
	Gc	188.1 ± 4.3	174.6 ± 10.7	167.7 ± 7.1	170.9 ± 2.7	287.1 ± 24.6	268.5 ± 137.1	—	—
	Dhcr7	365.7 ± 49.4	362.1 ± 8	343.9 ± 8.2	342.3 ± 10.8	281.7 ± 34.5	205.6 ± 60.3	—	—
	Rxra	226.4 ± 10.2	223 ± 1.8	223.7 ± 3.5	204 ± 7.3	314.3 ± 40.2	382.8 ± 51.2	—	—
	Rgn	189.6 ± 2.1	197.9 ± 2.4	184.4 ± 9.6	189.7 ± 6	245.3 ± 67.5	239.8 ± 71	—	—
	Slc23a1	193.9 ± 11.1	194.3 ± 5.7	192.5 ± 11.9	199.6 ± 3	252.5 ± 37.7	253.8 ± 72.6	—	—
	Slc23a2	423 ± 46.1	406 ± 23.9	503.9 ± 63	472.9 ± 15.8	266.5 ± 50	256.9 ± 137	—	—
	Gulo	187.3 ± 15	183 ± 3	181.2 ± 7.8	176 ± 3.2	269.8 ± 14.2	284.9 ± 81.8	—	—
Muscle	Cyp2r1	193.7 ± 12.3	191.8 ± 28	156.8 ± 4.3	157.4 ± 1.3	156.5 ± 0.6	147.7 ± 1.7	—	—
	Rxra	320.3 ± 17.5	339.4 ± 31.2	379.5 ± 72.1	365.5 ± 8.5	262.3 ± 18.6	255.7 ± 19.5	—	—
	Slc23a2	201.2 ± 8	173 ± 5.3	159.1 ± 11	157.5 ± 3.8	154 ± 5.9	151 ± 7.8	—	—
	Gulo	173.9 ± 8.1	183 ± 39.5	159 ± 0.3	151.4 ± 3.3	151.6 ± 5	147.7 ± 4.8	—	—
Femur (6 weeks mice)	Vdr	—	—	420.5	658.6	547.1	1189.7	−0.6	−0.8
	Vdr	—	—	335.5	430.0	158.3	883.9	1.0	−1.1
	Gc	—	—	4648.7	785.7	1482.8	1028.4	1.6	−0.5
	Rgn	—	—	855.6	161.5	743.7	456.9	0.2	−1.0
Liver	Gc	62635.6	—	—	—	49704.3	—	0.4	—
	Dhcr7	3282.4	—	—	—	2753.2	—	0.4	—
	Rgn	40842.4	—	—	—	31661.3	—	0.3	—
	Slc23a2	2296.0	—	—	—	1259.1	—	0.7	—

**Figure 6:**
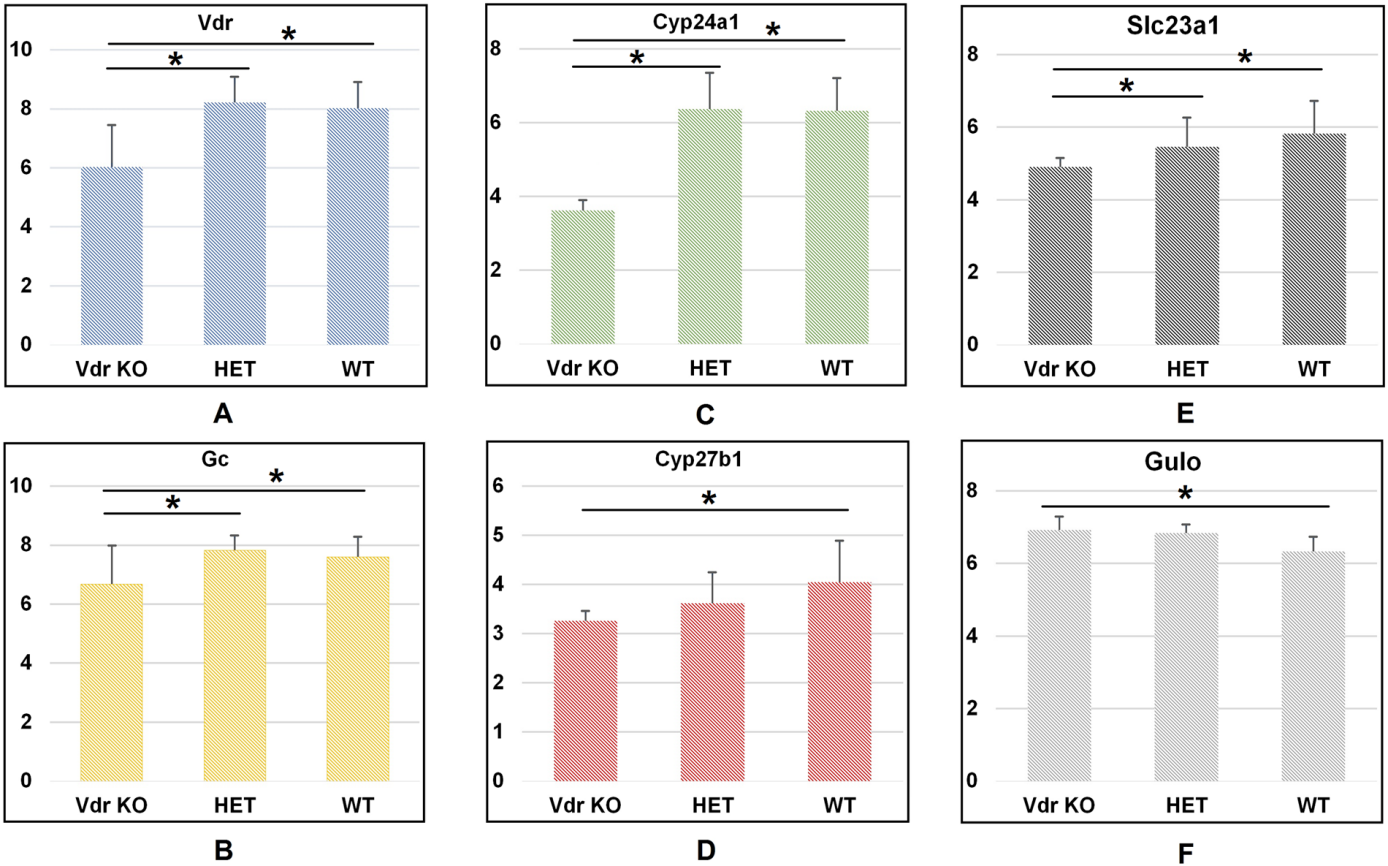
Different expression levels of VC and VD key genes in Vdr knockout mice group, Vdr heterozygous mice group, and wildtype mice group. *Represents P < 0.05. Numbers on the vertical lines indicate the relative expression level of genes.

For the analysis of data of *Vdr* knockout mice, we downloaded the database (details described in methods) and sorted the different expression results in the key genes of VC and VD. As can be seen in Fig. 6, when Vdr was knocked out, the expression levels of the associated gene were altered, including *Cyp24a1*, *Gc*, and *Cyp27b1*. In addition, the expression levels of *Slc23a1* and *Gulo* also showed significant differences when compared between the *Vdr* knockout group and the wildtype group (P < 0.05). The expression levels of those key genes are not affected in arthritic mice models with the non-mutated *Gulo* gene when compared to healthy control mice (Supplementary Table S1). These results support our hypothesis that the expression levels of VC and VD key genes influence each other.

## Discussion

**Figure 7:**
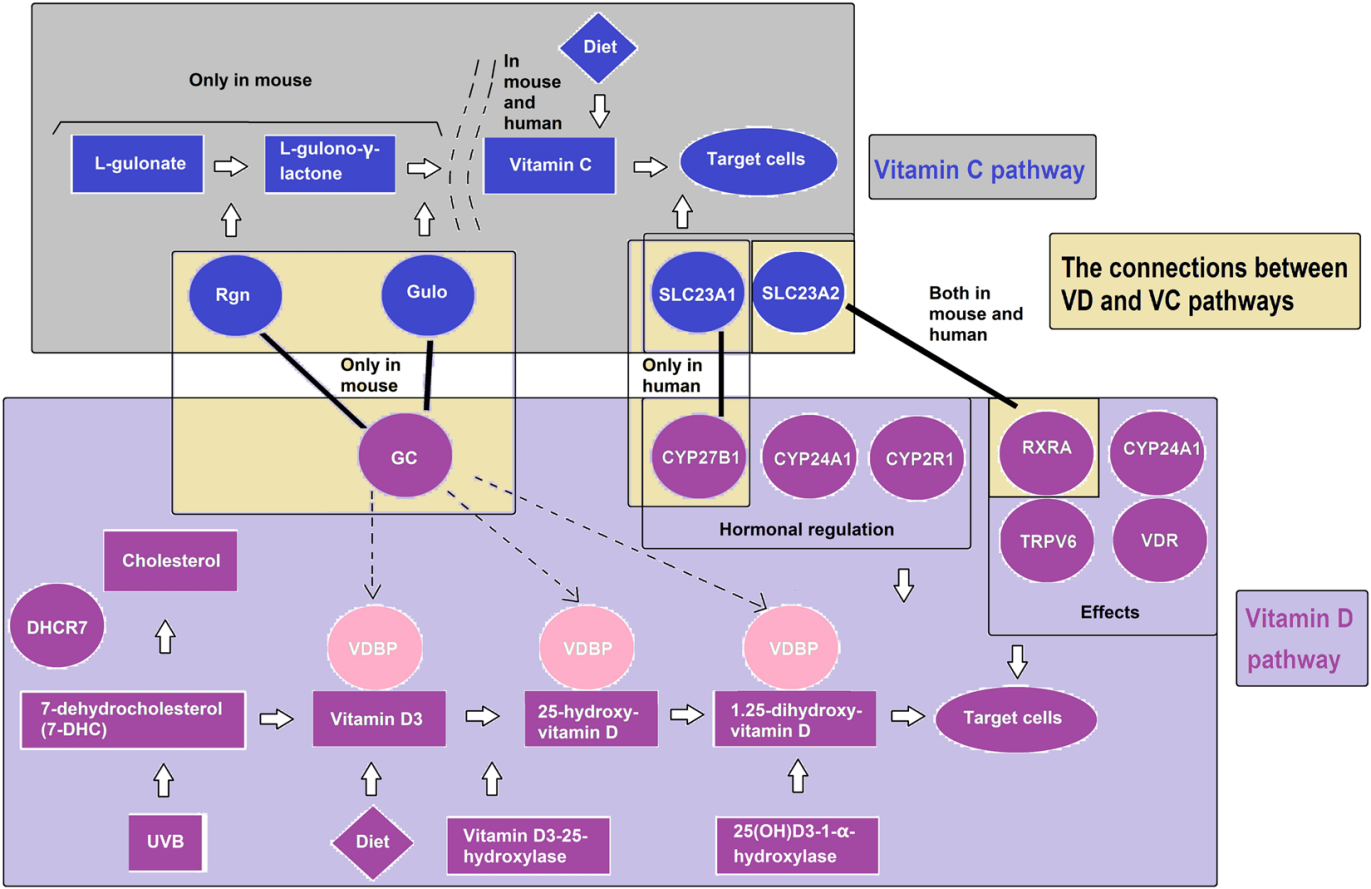
A hypothesis about the overlap between VC and VD synthesis and transport pathways. The VC and VD pathways are shown at the top and bottom of the figure while the interactions are shown in the middle. The black bold lines represent the VC and VD interaction.

Our data reveals for the first time the potentially key switch in the relationship between VC and VD pathways. To illustrate such an overlap, we have drawn a pathway diagram (Fig. 7). In Fig. 7, the upper part of the figure shows the VC synthesis and transport pathway. In mice and many other animals, it is through *Rgn* and *Gulo*, by which L-gulonate becomes L-gulono-γ-lactone and finally becomes VC. *Slc23a1* and *Slc23a2* are transporters for VC *in vivo*.

VD synthesis and transport pathways are illustrated in the lower part of Fig. 7. Vitamin D3 from UVB and diet are bound by VDBP until transport to target cells. VDBP is encoded by *Gc*. In the kidney, hormonal regulation significantly impacts the VD pathway and *CYP27B1*, *CYP24A1*, *CYP2R1*. *RXRA*, *CYP24A1*, *VDR,* and *TRPV6* are all functional in VD target cells.

In the middle part of Fig. 7 we illustrate how these two pathways are possibly connected. Our results show that *Rgn* is connected with *Gc* only in mice and *SLC23A1* is connected with *CYP27B1* only in humans. *Gulo* weakly connects with *Gc* in mice. *Slc23a2* connects with *RXRA* both in mouse and human. There may be some unknown genes involved in regulation, leading to their association.

A series of reported evidence supports our views. A recent study showed that plasma levels of vitamin D correlated with those of vitamin E and vitamin C^17^. Da Costa *et al*. reported levels of vitamin D-binding protein were inversely associated with levels of ascorbic acid in humans when they focused on identifying plasma proteins associated with circulating levels of ascorbic acid^18^. Our data also suggest that the associations between VD and VC can be direct or indirect. The direct association may be linked by *Rgn* and *Gulo*, *Gc* and *Rgn* in mouse, or *SLC23A1* and *CYP27B1* in humans. *SLC23A1* and *CYP27B1* are both highly expressed in kidney, and they also show a linkage in other tissues. The indirect associations could be through multiple ways connected by some other related genes, as we showed in the top 100 closely correlated genes to them, which need to be further studied.

A critical issue is that different individuals may utilize VD or VC with different efficiency, as the pathways are not independent. Our data may partially clarify why results of studies on the effects of VD and VC vary, i.e., because one element is affected by the other^19^. Our data may also explain why the value of VD deficiency thresholds (in measured by 25(OH)D) also varies^19–21^. Similarly, recommended optimal VC and VD levels have also generally been independent, without considering possible interactions.

Equally important, VC and VD have been used in combination with other agents in the treatment of disease^22–25^. According to our data, the optimal amount of VC or VD when used in treatment should be based on the balanced levels of both VC and VD. It is understandable that results of different combinations among drugs and VC or VD may also differ due to particular interactions between the drug and VC or VD. It is necessary to investigate whether it is optimal to add other vitamins to work synergistically to produce a better therapeutic effect.

The key genes in the connection between VC and VD may be used as alternative targets in disease treatment, nutrition, and supplementation. In addition to their function in VC and VD pathways, these genes have other important functions^26,27^. When these genes are utilized, their association may influence each other, which in turn, may affect the VC and VD pathway. Thus, a much broader effect of these genes should be considered when they are used for other purposes.

In this article, we focus on the mRNA expression level or genomic levels, not on protein levels. However, with multiple analyses with different methodologies and at different levels, we have found evidence to demonstrate that some overlap exists between VC and VD synthesis and transport pathways. It is clear that additional studies such as at protein levels should be conducted in the future. Our study has several limitations. First of all, most of our analyses are based on mouse models, or gene expressions profiles at one time point. The human sample is from the Genotype-Tissue Expression (GTEx) project, with a sample of around 500 that was 85% Caucasian. Secondly, we did not test for potential sex differences. Thirdly, the mouse data are mainly from BXD RI strains, which were derived from two parental strains, (C57B6/J and DBA/2 J), and thus may not represent all mouse strains. Furthermore, our analysis is only based on the association of the expression levels between genes. Influence by polymorphism and other epidermal factors was not been considered. Further studies on verification of these results and further analysis of potential age and sex differences will advance research into mechanisms of VD and VC effects.

In summary, our study suggests that in mice, the VC and VD functional pathways are connected mainly through *Gc*-*Rgn* and *Gc*-*Gulo*. In humans, the connection is mainly through *SLC23A1* and *CYP27B1*. The significant differences raise the question of whether it is suitable to conduct research using VC endogenous production rodent models. The association between VD and VC pathways indicates a need for both to be considered in their nutritional and therapeutic applications.

## Methods

### Data source

All the relevant data were derived from GeneNetwork which is publicly accessible via http://www.GeneNetwork.org. We separately analyzed BXD strains’ whole genome expression profile in spleen (UTHSC Affy MoGene 1.0 ST Spleen (Dec10) RMA), liver (GSE16780 UCLA Hybrid MDP Liver Affy HT M430A (Sep11) RMA)^28^, lung (HZI Lung M430v2 (Apr08) RMA), and kidney (Mouse kidney M430v2 Sex Balanced (Aug06) RMA). These four sets of data using the GeneNetwork platform were the best resource for us to explore whether VC and VD have relationships. For humans, we chose whole genome expression data at GTEx V5 group in GeneNetwork and chose the same tissues as we have used in mice^29,30^.

Sfx mice were used to validate the connection of key genes in the VC and VD pathways. The key gene in VC synthesis, Gulo, was mutated in the Sfx mice. Because Sfx mice are under Balb/c background, Balb/c mice were used as the control group. The microarray data has been reported in previous studies^31,32^. RNA extract and data analysis followed our previous procedures^31^. These experiments were designed to compare the expression level in Sfx mice with/without VC treatment and in Balb/c mice, and the tissues included liver, femur, and muscle. Real-time PCR was applied to validate the microarray results.

The data from a public database in NCBI GEO DataSets (GSE61583) was used to confirm that VC genes are affected when Vdr was knocked out. The study is an analysis of mouse placenta retrieved at day 18.5 pc with Vdr gene knockout. (https://www.ncbi.nlm.nih.gov/geo/query/acc.cgi?acc=GSE61583). Mice were divided into three groups: Vdr knockout mice group, Vdr heterozygous mice group, and wildtype mice group^33,34^.

### Gene network construction

The graphic application tools in GeneNetwork can construct key genes of VC and VD pathways. We chose Spring Model layout (energy reduction) for all graphic subjects. Different correlations were drawn in different colors which are the same as in our previous publication^16^. We separately analyzed Pearson, Literature, and Tissue in different tissues of mouse and human.

### eQTL mapping

We used the standard resolution provided by GeneNetwork to identify eQTL position. Interval mapping tools mapped each chromosome with 1000 permutation tests to compute eQTLs. Expression values from different strains are considered to be phenotypes. A molecular marker along the chromosome was used as the genotype of the position on the chromosome. Then, the expression value of the probability of a particular genotype at the test position between the two flanking markers was compared. Statistical probability was used to assess the significance of the eQTL at a location to ultimately produce an eQTL^16^.

### Correlation between *Gc* and *Rgn*

The sample correlation is computed between expression levels of *Gc* or *Rgn* and any other traits in the sample database selected. Pearson’s Rank was chosen when the sample size is large. Spearman’s Rank was used when the sample size was small (<20) or when there are influential outliers. We separately computed the top 100 probes for *Gc* and *Rgn* in each tissue.

### Statistical analysis

R values and P values were obtained from GeneNetwork automatically. We used standard criteria to categorize the strength of the correlation. Thus, when the R value was equal to or more than 0.7 or −0.7, we regarded the correlation as a strong positive or negative index. An R value of between 0.35 and 0.69 or between −0.35 and −0.69 was considered a weak correlation. We treated R values between 0 and 0.35 or between 0 and −0.35 as no correlation^35^.

## Supplementary information

Supplementary materials

## Data Availability

The authors declare that the data will be available without restrictions.
