# Supplementary material for: Multiple genome analyses reveal key genes in Vitamin C and Vitamin D synthesis and transport pathways are shared
Source: Sci Rep. 2019 Nov 14;9:16811. doi: 10.1038/s41598-019-53074-9 (PMC6856197; doi:10.1038/s41598-019-53074-9)
Supplement: Supplementary file 1 — Supplementary materials [file 41598_2019_53074_MOESM1_ESM.docx]

**Multiple genome analyses reveal key genes in Vitamin C and Vitamin D synthesis and transport pathways are shared**

Wei Dong^1,2^, Cheng Tian^1^, Yan Jiao^1^, Savannah Blackwell^1^, Ge Lou^2^, Arnold Postlethwaite^3^, Weikuan Gu^1,4,^*, Dianjun Sun^5^*

^1^ Department of Orthopaedic Surgery and Biomedical Engineering, University of Tennessee Health Science Center (UTHSC), 956 Court Av, Memphis, TN 38163, USA

^2^ Department of Gynecology, Harbin Medical University Cancer Hospital, No. 8 building, Haping Road, Harbin, Heilongjiang 150001, China

^3^ Department of Medicine, University of Tennessee Health Science Center (UTHSC), 956 Court Av, Memphis, TN 38163, USA

^4^ Research Service, Veterans Affairs Medical Center, 1030 Jefferson Avenue, Memphis, TN 38104, USA

^5^ Center for Endemic Disease Control, Chinese Center for Disease Control and Prevention, Harbin Medical University; Key Laboratory of Etiologic Epidemiology, Education Bureau of Heilongjiang Province & Ministry of Health (23618104), 157 Baojian Road, Harbin, Heilongjiang 150081, China

* Corresponding author

E-mail: wgu@uthsc.edu (WG); hrbmusdj@163.com (DS)

**Supplementary Materials**

**Supplementary Table S1. The expression levels of key genes of VC and VD in normal mice and arthritis mouse models.** WT represents wild type mice. Balb/c KO represents Interleukin-1 receptor antagonist knockout Balb/c mice commonly used as an arthritis model. DBA KO represents Interleukin-1 receptor antagonist DBA knockout mice which is not associated with arthritis. There are no differences among the wildtype mouse model, the arthritis model, and the gene knockout model.

| **SYMBOL** | **Balb/c-WT** | **DBA-WT** | **Balb/c-KO** | **DBA-KO** |  |
| --- | --- | --- | --- | --- | --- |
| **Vdr** | **140.13** | **152.16** | **143.58** | **144.42** |  |
| **Gc** | **103.12** | **94.92** | **95.04** | **94.48** |  |
| **Cyp2r1** | **117.47** | **108.89** | **106.87** | **114.88** |  |
| **Cyp24a1** | **117.37** | **123.74** | **109.91** | **122.34** |  |
| **Cyp27b1** | **92.94** | **91.11** | **90.57** | **94.65** |  |
| **Dhcr7** | **230.52** | **230.51** | **244.21** | **231.27** |  |
| **Trpv6** | **105.33** | **122.04** | **109.99** | **106.24** |  |
| **Rxra** | **150.2** | **142.92** | **149.99** | **167.12** |  |
| **Rgn** | **101.33** | **96.34** | **96.76** | **104.6** |  |
| **Slc23a1** | **107** | **113.47** | **105.37** | **103.24** |  |
| **Slc23a2** | **511** | **505.79** | **490.23** | **511.84** |  |
| **Gulo** | **103.82** | **96.88** | **100.06** | **97.25** |  |
|  |  |  |  |  |  |

**Supplementary Table S2. Comparing of 100 top genes closely related to Il1rn, Avpr1a, Vdr, and Lep in lung of mice.**

| BXD lung Pearson’s coefficient | |  |  |  |  |  |  |  |
| --- | --- | --- | --- | --- | --- | --- | --- | --- |
| **Symbol** | **Symbol** |  |  | **Symbol** | **Symbol** |  |  |  |
| **Il1rn** | **Avpr1a** | #N/A |  | **Il1rn** | **Vdr** | #N/A |  |  |
| Tpd52 | Avpr1a | #N/A |  | Tpd52 | Vav3 | #N/A |  |  |
| Csf2ra | Per3 | #N/A |  | Csf2ra | Sccpdh | #N/A |  |  |
| Slc31a2 | 1110065D03Rik | #N/A |  | Slc31a2 | Cotl1 | #N/A |  |  |
| Asb13 | Arntl | #N/A |  | Asb13 | Mpdz | #N/A |  |  |
| Cd200r1 | Dtx4 | #N/A |  | Cd200r1 | Zfp37 | #N/A |  |  |
| Mrpl9 | Hlf | #N/A |  | Mrpl9 | Cln6 | #N/A |  |  |
| Pldn | Per3 | #N/A |  | Pldn | Nup54 | #N/A |  |  |
| Clec5a | Dbp | #N/A |  | Clec5a | Cit | #N/A |  |  |
| Lcp1 | Dbp | #N/A |  | Lcp1 | Irs2 | #N/A |  |  |
| Prps2 | Per3 | #N/A |  | Prps2 | Tnks1bp1 | #N/A |  |  |
| Atg2b | Dtx4 | #N/A |  | Atg2b | Nfix | #N/A |  |  |
| Snap29 | Tspan4 | #N/A |  | Snap29 | C3orf67 | #N/A |  |  |
| Crsp2 | Tef | #N/A |  | Crsp2 | Ii | #N/A |  |  |
| Capza2 | Zfp346 | #N/A |  | Capza2 | 0610008F07Rik | #N/A |  |  |
| Txnip | 1810054O13Rik | #N/A |  | Txnip | Spint1 | #N/A |  |  |
| Plekha2 | Aco2 | #N/A |  | Plekha2 | Cotl1 | #N/A |  |  |
| Ednra | 4933427D06 | #N/A |  | Ednra | Laptm5 | #N/A |  |  |
| Muc6 | Hlf | #N/A |  | Muc6 | Tbc1d12 | #N/A |  |  |
| Bicd2 | Npas2 | #N/A |  | Bicd2 | Sidt2 | #N/A |  |  |
| Ubr2 | Gm129 | #N/A |  | Ubr2 | Serpinf2 | #N/A |  |  |
| Ndst1 | Msc | #N/A |  | Ndst1 | 1700110N18Rik | #N/A |  |  |
| Ltbp1 | Gpr155 | #N/A |  | Ltbp1 | Prcp | #N/A |  |  |
| Il13ra1 | Per2 | #N/A |  | Il13ra1 | Itpr3 | #N/A |  |  |
| Ncf1 | Pik3ip1 | #N/A |  | Ncf1 | Zcsl2 | #N/A |  |  |
| Atf2 | Aldh2 | #N/A |  | Atf2 | Stx18 | #N/A |  |  |
| Cldn1 | Gm185 | #N/A |  | Cldn1 | Bteb1 | #N/A |  |  |
| Leprotl1 | 5430411C19Rik | #N/A |  | Leprotl1 | Sh3bp1 | #N/A |  |  |
| M6pr | Nfil3 | #N/A |  | M6pr | Foxp4 | #N/A |  |  |
| Pafah1b2 | Spon2 | #N/A |  | Pafah1b2 | Top2b | #N/A |  |  |
| A630082K20Rik | Dapk1 | #N/A |  | A630082K20Rik | Dnm1l | #N/A |  |  |
| Cript | Nr1d2 | #N/A |  | Cript | Klhdc2 | #N/A |  |  |
| Ssu72 | Centa2 | #N/A |  | Ssu72 | Snrp70 | #N/A |  |  |
| Mrc2 | Cer1 | #N/A |  | Mrc2 | Arhgap27 | #N/A |  |  |
| Sypl | Prdx6 | #N/A |  | Sypl | 2900016B01Rik | #N/A |  |  |
| Kifap3 | Ppp1r3c | #N/A |  | Kifap3 | Iqce | #N/A |  |  |
| Rp2 | Kiaa1737 | #N/A |  | Rp2 | D830039M14Rik | #N/A |  |  |
| Mpzl2 | Bhlhe40 | #N/A |  | Mpzl2 | Man2b1 | #N/A |  |  |
| Atxn2 | Wee1 | #N/A |  | Atxn2 | B230217C12Rik | #N/A |  |  |
| Ndfip1 | Rev1l | #N/A |  | Ndfip1 | Cfl2 | #N/A |  |  |
| 1600029D21Rik | Adm | #N/A |  | 1600029D21Rik | Tial1 | #N/A |  |  |
| Snu13 | Adm | #N/A |  | Snu13 | 1700022L09Rik | #N/A |  |  |
| Rap1b | Ahcyl2 | #N/A |  | Rap1b | AI449175 | #N/A |  |  |
| Mtpn | Paip2 | #N/A |  | Mtpn | Aldoc | #N/A |  |  |
| Cyld | Rhebl1 | #N/A |  | Cyld | Mrg1 | #N/A |  |  |
| Dnajc24 | Wee1 | #N/A |  | Dnajc24 | Zfp647 | #N/A |  |  |
| Plek | Heatr1 | #N/A |  | Plek | Denr | #N/A |  |  |
| Fcgr2b | Hdhd4 | #N/A |  | Fcgr2b | Cotl1 | #N/A |  |  |
| Rab2a | Per2 | #N/A |  | Rab2a | Stxbp6 | #N/A |  |  |
| Cd53 | Neu1 | #N/A |  | Cd53 | Gipc3 | #N/A |  |  |
| Lrrc59 | Lphn3 | #N/A |  | Lrrc59 | Itih4 | #N/A |  |  |
| Sla | Ankrd12 | #N/A |  | Sla | Mrps22 | #N/A |  |  |
| Srd5a2l | 5430416O09Rik | #N/A |  | Srd5a2l | Klhdc2 | #N/A |  |  |
| Gng2 | Angptl2 | #N/A |  | Gng2 | Cotl1 | #N/A |  |  |
| Bptf | Cxcl15 | #N/A |  | Bptf | B230373P09Rik | #N/A |  |  |
| Nsun3 | 9230106B05Rik | #N/A |  | Nsun3 | AK144361 | #N/A |  |  |
| Gng2 | Serpine1 | #N/A |  | Gng2 | Smap2 | #N/A |  |  |
| Il13ra1 | 2900001A12Rik | #N/A |  | Il13ra1 | Paxip1 | #N/A |  |  |
| Ugcg | Ankrd12 | #N/A |  | Ugcg | LOC378878 | #N/A |  |  |
| Ash1l | Star | #N/A |  | Ash1l | Clasp2 | #N/A |  |  |
| G630041M05Rik | Wee1 | #N/A |  | G630041M05Rik | Gpaa1 | #N/A |  |  |
| St3gal2 | Ccbe1 | #N/A |  | St3gal2 | Prdm16 | #N/A |  |  |
| AF119384 | Rnf112 | #N/A |  | AF119384 | Galgt1 | #N/A |  |  |
| Ccl9 | Lonrf1 | #N/A |  | Ccl9 | Cmip | #N/A |  |  |
| Smap | Il18r1 | #N/A |  | Smap | 4930540E01Rik | #N/A |  |  |
| Dck | Ccnjl | #N/A |  | Dck | 2010001J22Rik | #N/A |  |  |
| Ppt1 | AU041783 | #N/A |  | Ppt1 | Pde7b | #N/A |  |  |
| Ncf1 | Clock | #N/A |  | Ncf1 | Nfix | #N/A |  |  |
| Sar1a | Usp2 | #N/A |  | Sar1a | Mnt | #N/A |  |  |
| Tfrc | Lonrf3 | #N/A |  | Tfrc | Ptbp2 | #N/A |  |  |
| Sri | 2610207P08Rik | #N/A |  | Sri | Syt13 | #N/A |  |  |
| Cdgap | Spon2 | #N/A |  | Cdgap | 4432409M07Rik | #N/A |  |  |
| Dlgap4 | Mthfd1l | #N/A |  | Dlgap4 | Mlc1 | #N/A |  |  |
| Ppm1a | Slc25a33 | #N/A |  | Ppm1a | 5730457F11Rik | #N/A |  |  |
| Serp1 | Panx3 | #N/A |  | Serp1 | Catna3 | #N/A |  |  |
| Gosr1 | Clock | #N/A |  | Gosr1 | Tapbp | #N/A |  |  |
| 2810012G03Rik | Usp2 | #N/A |  | 2810012G03Rik | Tsta3 | #N/A |  |  |
| Pycr1 | Per2 | #N/A |  | Pycr1 | Centb1 | #N/A |  |  |
| Sgcz | Adamts4 | #N/A |  | Sgcz | B130034C11Rik | #N/A |  |  |
| Ywhae | 2310004L02Rik | #N/A |  | Ywhae | Tbc1d9 | #N/A |  |  |
| Prps2 | St6galnac2 | #N/A |  | Prps2 | Zfp533 | #N/A |  |  |
| Nars | Nr1d1 | #N/A |  | Nars | Ntn3 | #N/A |  |  |
| Pdpr | Rps6ka3 | #N/A |  | Pdpr | Ube1c | #N/A |  |  |
| Nus1 | Tulp3 | #N/A |  | Nus1 | Kcnab2 | #N/A |  |  |
| Tmed10 | Spic | #N/A |  | Tmed10 | Snapc2 | #N/A |  |  |
| Sipa1l2 | Clock | #N/A |  | Sipa1l2 | Nap1l5 | #N/A |  |  |
| Slc35a3 | Clock | #N/A |  | Slc35a3 | Pak7 | #N/A |  |  |
| Csnrp2 | LOC193217 | #N/A |  | Csnrp2 | Rpl14 | #N/A |  |  |
| Lgals8 | 1810019D21Rik | #N/A |  | Lgals8 | Rala | #N/A |  |  |
| Pum1 | Suox | #N/A |  | Pum1 | Azi2 | #N/A |  |  |
| Cd44 | 1110007C24Rik | #N/A |  | Cd44 | Pld3 | #N/A |  |  |
| 1200011I18Rik | Klhl13 | #N/A |  | 1200011I18Rik | Ankrd29 | #N/A |  |  |
| Limd1 | Npas2 | #N/A |  | Limd1 | Aytl2 | #N/A |  |  |
| Irak3 | Gpr146 | #N/A |  | Irak3 | Tnrc18 | #N/A |  |  |
| Fundc1 | Vtcn1 | #N/A |  | Fundc1 | 8430438D04Rik | #N/A |  |  |
| 5830411E10Rik | Pfkfb3 | #N/A |  | 5830411E10Rik | Ttc7 | #N/A |  |  |
| Stard5 | 2410043F08Rik | #N/A |  | Stard5 | Kif21b | #N/A |  |  |
| Ppp1cc | Sytl2 | #N/A |  | Ppp1cc | Gpr132 | #N/A |  |  |
| Arhgap30 | AB041803 | #N/A |  | Arhgap30 | LOC224833 | #N/A |  |  |
| Mr1 | Stx6 | #N/A |  | Mr1 | Arhgef1 | #N/A |  |  |
|  |  |  |  |  |  |  |  |  |
|  |  |  |  |  |  |  |  |  |
| **Symbol** | **Symbol** |  |  | **Symbol** | **Symbol** |  |  |  |
| **Avpr1a** | **Vdr** | #N/A |  | **Avpr1a** | **Lep** | #N/A |  |  |
| Avpr1a | Vav3 | #N/A |  | Avpr1a | Pnpla3 | #N/A |  |  |
| Per3 | Sccpdh | #N/A |  | Per3 | 2310076O14Rik | #N/A |  |  |
| 1110065D03Rik | Cotl1 | #N/A |  | 1110065D03Rik | Plin | #N/A |  |  |
| Arntl | Mpdz | #N/A |  | Arntl | Sncg | #N/A |  |  |
| Dtx4 | Zfp37 | #N/A |  | Dtx4 | Aoc3 | #N/A |  |  |
| Hlf | Cln6 | #N/A |  | Hlf | Ptger3 | #N/A |  |  |
| Per3 | Nup54 | #N/A |  | Per3 | LOC330189 | #N/A |  |  |
| Dbp | Cit | #N/A |  | Dbp | Ptger3 | #N/A |  |  |
| Dbp | Irs2 | #N/A |  | Dbp | Thbs2 | #N/A |  |  |
| Per3 | Tnks1bp1 | #N/A |  | Per3 | Lgals12 | #N/A |  |  |
| Dtx4 | Nfix | #N/A |  | Dtx4 | Paqr9 | #N/A |  |  |
| Tspan4 | C3orf67 | #N/A |  | Tspan4 | S3-12 | #N/A |  |  |
| Tef | Ii | #N/A |  | Tef | Mmd | #N/A |  |  |
| Zfp346 | 0610008F07Rik | #N/A |  | Zfp346 | Car3 | #N/A |  |  |
| 1810054O13Rik | Spint1 | #N/A |  | 1810054O13Rik | Dbi | #N/A |  |  |
| Aco2 | Cotl1 | #N/A |  | Aco2 | Orm1 | #N/A |  |  |
| 4933427D06 | Laptm5 | #N/A |  | 4933427D06 | A530053G22Rik | #N/A |  |  |
| Hlf | Tbc1d12 | #N/A |  | Hlf | Cspg3 | #N/A |  |  |
| Npas2 | Sidt2 | #N/A |  | Npas2 | Pgm2 | #N/A |  |  |
| Gm129 | Serpinf2 | #N/A |  | Gm129 | Mlxipl | #N/A |  |  |
| Msc | 1700110N18Rik | #N/A |  | Msc | Ebf3 | #N/A |  |  |
| Gpr155 | Prcp | #N/A |  | Gpr155 | Cspg3 | #N/A |  |  |
| Per2 | Itpr3 | #N/A |  | Per2 | Msr2 | #N/A |  |  |
| Pik3ip1 | Zcsl2 | #N/A |  | Pik3ip1 | Itih5 | #N/A |  |  |
| Aldh2 | Stx18 | #N/A |  | Aldh2 | Agpat2 | #N/A |  |  |
| Gm185 | Bteb1 | #N/A |  | Gm185 | Mmd | #N/A |  |  |
| 5430411C19Rik | Sh3bp1 | #N/A |  | 5430411C19Rik | Thrsp | #N/A |  |  |
| Nfil3 | Foxp4 | #N/A |  | Nfil3 | Pparg | #N/A |  |  |
| Spon2 | Top2b | #N/A |  | Spon2 | 9430028I06Rik | #N/A |  |  |
| Dapk1 | Dnm1l | #N/A |  | Dapk1 | Tusc5 | #N/A |  |  |
| Nr1d2 | Klhdc2 | #N/A |  | Nr1d2 | Fabp4 | #N/A |  |  |
| Centa2 | Snrp70 | #N/A |  | Centa2 | Fabp4 | #N/A |  |  |
| Cer1 | Arhgap27 | #N/A |  | Cer1 | Spata18 | #N/A |  |  |
| Prdx6 | 2900016B01Rik | #N/A |  | Prdx6 | Fabp4 | #N/A |  |  |
| Ppp1r3c | Iqce | #N/A |  | Ppp1r3c | Sucnr1 | #N/A |  |  |
| Kiaa1737 | D830039M14Rik | #N/A |  | Kiaa1737 | BC034068 | #N/A |  |  |
| Bhlhe40 | Man2b1 | #N/A |  | Bhlhe40 | Immp2l | #N/A |  |  |
| Wee1 | B230217C12Rik | #N/A |  | Wee1 | 4933403F05Rik | #N/A |  |  |
| Rev1l | Cfl2 | #N/A |  | Rev1l | Tkt | #N/A |  |  |
| Adm | Tial1 | #N/A |  | Adm | Amy1 | #N/A |  |  |
| Adm | 1700022L09Rik | #N/A |  | Adm | Sde2 | #N/A |  |  |
| Ahcyl2 | AI449175 | #N/A |  | Ahcyl2 | 2400009B08Rik | #N/A |  |  |
| Paip2 | Aldoc | #N/A |  | Paip2 | Cidec | #N/A |  |  |
| Rhebl1 | Mrg1 | #N/A |  | Rhebl1 | Gcm2 | #N/A |  |  |
| Wee1 | Zfp647 | #N/A |  | Wee1 | Tshr | #N/A |  |  |
| Heatr1 | Denr | #N/A |  | Heatr1 | Mc2r | #N/A |  |  |
| Hdhd4 | Cotl1 | #N/A |  | Hdhd4 | Slc16a1 | #N/A |  |  |
| Per2 | Stxbp6 | #N/A |  | Per2 | A230106N23 | #N/A |  |  |
| Neu1 | Gipc3 | #N/A |  | Neu1 | Thrsp | #N/A |  |  |
| Lphn3 | Itih4 | #N/A |  | Lphn3 | Dgat2 | #N/A |  |  |
| Ankrd12 | Mrps22 | #N/A |  | Ankrd12 | Prim2 | #N/A |  |  |
| 5430416O09Rik | Klhdc2 | #N/A |  | 5430416O09Rik | Stk19 | #N/A |  |  |
| Angptl2 | Cotl1 | #N/A |  | Angptl2 | 1110032E23Rik | #N/A |  |  |
| Cxcl15 | B230373P09Rik | #N/A |  | Cxcl15 | 2610001E17Rik | #N/A |  |  |
| 9230106B05Rik | AK144361 | #N/A |  | 9230106B05Rik | Gpd2 | #N/A |  |  |
| Serpine1 | Smap2 | #N/A |  | Serpine1 | 4933430A09 | #N/A |  |  |
| 2900001A12Rik | Paxip1 | #N/A |  | 2900001A12Rik | Ebf2 | #N/A |  |  |
| Ankrd12 | LOC378878 | #N/A |  | Ankrd12 | Aps | #N/A |  |  |
| Star | Clasp2 | #N/A |  | Star | Sycp3 | #N/A |  |  |
| Wee1 | Gpaa1 | #N/A |  | Wee1 | 4930429N05Rik | #N/A |  |  |
| Ccbe1 | Prdm16 | #N/A |  | Ccbe1 | Dbi | #N/A |  |  |
| Rnf112 | Galgt1 | #N/A |  | Rnf112 | Lgals12 | #N/A |  |  |
| Lonrf1 | Cmip | #N/A |  | Lonrf1 | Btc | #N/A |  |  |
| Il18r1 | 4930540E01Rik | #N/A |  | Il18r1 | Snf1lk2 | #N/A |  |  |
| Ccnjl | 2010001J22Rik | #N/A |  | Ccnjl | Gpr1 | #N/A |  |  |
| AU041783 | Pde7b | #N/A |  | AU041783 | Folr2 | #N/A |  |  |
| Clock | Nfix | #N/A |  | Clock | Ybx2 | #N/A |  |  |
| Usp2 | Mnt | #N/A |  | Usp2 | Amacr | #N/A |  |  |
| Lonrf3 | Ptbp2 | #N/A |  | Lonrf3 | Usp8 | #N/A |  |  |
| 2610207P08Rik | Syt13 | #N/A |  | 2610207P08Rik | Pcdh7 | #N/A |  |  |
| Spon2 | 4432409M07Rik | #N/A |  | Spon2 | Aqp7 | #N/A |  |  |
| Mthfd1l | Mlc1 | #N/A |  | Mthfd1l | Ntrk3 | #N/A |  |  |
| Slc25a33 | 5730457F11Rik | #N/A |  | Slc25a33 | Smoc1 | #N/A |  |  |
| Panx3 | Catna3 | #N/A |  | Panx3 | Cacul1 | #N/A |  |  |
| Clock | Tapbp | #N/A |  | Clock | Pde10a | #N/A |  |  |
| Usp2 | Tsta3 | #N/A |  | Usp2 | Slc25a19 | #N/A |  |  |
| Per2 | Centb1 | #N/A |  | Per2 | Tshr | #N/A |  |  |
| Adamts4 | B130034C11Rik | #N/A |  | Adamts4 | Ndufab1 | #N/A |  |  |
| 2310004L02Rik | Tbc1d9 | #N/A |  | 2310004L02Rik | Adrb3 | #N/A |  |  |
| St6galnac2 | Zfp533 | #N/A |  | St6galnac2 | Gpd2 | #N/A |  |  |
| Nr1d1 | Ntn3 | #N/A |  | Nr1d1 | AI596198 | #N/A |  |  |
| Rps6ka3 | Ube1c | #N/A |  | Rps6ka3 | 2610034M16Rik | #N/A |  |  |
| Tulp3 | Kcnab2 | #N/A |  | Tulp3 | Mapbpip | #N/A |  |  |
| Spic | Snapc2 | #N/A |  | Spic | AW491448 | #N/A |  |  |
| Clock | Nap1l5 | #N/A |  | Clock | Brp44 | #N/A |  |  |
| Clock | Pak7 | #N/A |  | Clock | Csnk | #N/A |  |  |
| LOC193217 | Rpl14 | #N/A |  | LOC193217 | Pkd2l2 | #N/A |  |  |
| 1810019D21Rik | Rala | #N/A |  | 1810019D21Rik | Cox7a1 | #N/A |  |  |
| Suox | Azi2 | #N/A |  | Suox | Cd59a | #N/A |  |  |
| 1110007C24Rik | Pld3 | #N/A |  | 1110007C24Rik | Gpd1 | #N/A |  |  |
| Klhl13 | Ankrd29 | #N/A |  | Klhl13 | 6530401D17Rik | #N/A |  |  |
| Npas2 | Aytl2 | #N/A |  | Npas2 | Adipoq | #N/A |  |  |
| Gpr146 | Tnrc18 | #N/A |  | Gpr146 | C730029A08Rik | #N/A |  |  |
| Vtcn1 | 8430438D04Rik | #N/A |  | Vtcn1 | Sdhc | #N/A |  |  |
| Pfkfb3 | Ttc7 | #N/A |  | Pfkfb3 | 4930520O04Rik | #N/A |  |  |
| 2410043F08Rik | Kif21b | #N/A |  | 2410043F08Rik | Dlat | #N/A |  |  |
| Sytl2 | Gpr132 | #N/A |  | Sytl2 | 2010003K11Rik | #N/A |  |  |
| AB041803 | LOC224833 | #N/A |  | AB041803 | Slit2 | #N/A |  |  |
| Stx6 | Arhgef1 | #N/A |  | Stx6 | Pygl | #N/A |  |  |
|  |  |  |  |  |  |  |  |  |
| **Symbol** | **Symbol** |  |  | **Symbol** | **Symbol** |  |  |  |
| **Il1rn** | **Lep** | #N/A |  | **Vdr** | **Lep** | #N/A |  |  |
| Tpd52 | Pnpla3 | #N/A |  | Vav3 | Pnpla3 | #N/A |  |  |
| Csf2ra | 2310076O14Rik | #N/A |  | Sccpdh | 2310076O14Rik | #N/A |  |  |
| Slc31a2 | Plin | #N/A |  | Cotl1 | Plin | #N/A |  |  |
| Asb13 | Sncg | #N/A |  | Mpdz | Sncg | #N/A |  |  |
| Cd200r1 | Aoc3 | #N/A |  | Zfp37 | Aoc3 | #N/A |  |  |
| Mrpl9 | Ptger3 | #N/A |  | Cln6 | Ptger3 | #N/A |  |  |
| Pldn | LOC330189 | #N/A |  | Nup54 | LOC330189 | #N/A |  |  |
| Clec5a | Ptger3 | #N/A |  | Cit | Ptger3 | #N/A |  |  |
| Lcp1 | Thbs2 | #N/A |  | Irs2 | Thbs2 | #N/A |  |  |
| Prps2 | Lgals12 | #N/A |  | Tnks1bp1 | Lgals12 | #N/A |  |  |
| Atg2b | Paqr9 | #N/A |  | Nfix | Paqr9 | #N/A |  |  |
| Snap29 | S3-12 | #N/A |  | C3orf67 | S3-12 | #N/A |  |  |
| Crsp2 | Mmd | #N/A |  | Ii | Mmd | #N/A |  |  |
| Capza2 | Car3 | #N/A |  | 0610008F07Rik | Car3 | #N/A |  |  |
| Txnip | Dbi | #N/A |  | Spint1 | Dbi | #N/A |  |  |
| Plekha2 | Orm1 | #N/A |  | Cotl1 | Orm1 | #N/A |  |  |
| Ednra | A530053G22Rik | #N/A |  | Laptm5 | A530053G22Rik | #N/A |  |  |
| Muc6 | Cspg3 | #N/A |  | Tbc1d12 | Cspg3 | #N/A |  |  |
| Bicd2 | Pgm2 | #N/A |  | Sidt2 | Pgm2 | #N/A |  |  |
| Ubr2 | Mlxipl | #N/A |  | Serpinf2 | Mlxipl | #N/A |  |  |
| Ndst1 | Ebf3 | #N/A |  | 1700110N18Rik | Ebf3 | #N/A |  |  |
| Ltbp1 | Cspg3 | #N/A |  | Prcp | Cspg3 | #N/A |  |  |
| Il13ra1 | Msr2 | #N/A |  | Itpr3 | Msr2 | #N/A |  |  |
| Ncf1 | Itih5 | #N/A |  | Zcsl2 | Itih5 | #N/A |  |  |
| Atf2 | Agpat2 | #N/A |  | Stx18 | Agpat2 | #N/A |  |  |
| Cldn1 | Mmd | #N/A |  | Bteb1 | Mmd | #N/A |  |  |
| Leprotl1 | Thrsp | #N/A |  | Sh3bp1 | Thrsp | #N/A |  |  |
| M6pr | Pparg | #N/A |  | Foxp4 | Pparg | #N/A |  |  |
| Pafah1b2 | 9430028I06Rik | #N/A |  | Top2b | 9430028I06Rik | #N/A |  |  |
| A630082K20Rik | Tusc5 | #N/A |  | Dnm1l | Tusc5 | #N/A |  |  |
| Cript | Fabp4 | #N/A |  | Klhdc2 | Fabp4 | #N/A |  |  |
| Ssu72 | Fabp4 | #N/A |  | Snrp70 | Fabp4 | #N/A |  |  |
| Mrc2 | Spata18 | #N/A |  | Arhgap27 | Spata18 | #N/A |  |  |
| Sypl | Fabp4 | #N/A |  | 2900016B01Rik | Fabp4 | #N/A |  |  |
| Kifap3 | Sucnr1 | #N/A |  | Iqce | Sucnr1 | #N/A |  |  |
| Rp2 | BC034068 | #N/A |  | D830039M14Rik | BC034068 | #N/A |  |  |
| Mpzl2 | Immp2l | #N/A |  | Man2b1 | Immp2l | #N/A |  |  |
| Atxn2 | 4933403F05Rik | #N/A |  | B230217C12Rik | 4933403F05Rik | #N/A |  |  |
| Ndfip1 | Tkt | #N/A |  | Cfl2 | Tkt | #N/A |  |  |
| 1600029D21Rik | Amy1 | #N/A |  | Tial1 | Amy1 | #N/A |  |  |
| Snu13 | Sde2 | #N/A |  | 1700022L09Rik | Sde2 | #N/A |  |  |
| Rap1b | 2400009B08Rik | #N/A |  | AI449175 | 2400009B08Rik | #N/A |  |  |
| Mtpn | Cidec | #N/A |  | Aldoc | Cidec | #N/A |  |  |
| Cyld | Gcm2 | #N/A |  | Mrg1 | Gcm2 | #N/A |  |  |
| Dnajc24 | Tshr | #N/A |  | Zfp647 | Tshr | #N/A |  |  |
| Plek | Mc2r | #N/A |  | Denr | Mc2r | #N/A |  |  |
| Fcgr2b | Slc16a1 | #N/A |  | Cotl1 | Slc16a1 | #N/A |  |  |
| Rab2a | A230106N23 | #N/A |  | Stxbp6 | A230106N23 | #N/A |  |  |
| Cd53 | Thrsp | #N/A |  | Gipc3 | Thrsp | #N/A |  |  |
| Lrrc59 | Dgat2 | #N/A |  | Itih4 | Dgat2 | #N/A |  |  |
| Sla | Prim2 | #N/A |  | Mrps22 | Prim2 | #N/A |  |  |
| Srd5a2l | Stk19 | #N/A |  | Klhdc2 | Stk19 | #N/A |  |  |
| Gng2 | 1110032E23Rik | #N/A |  | Cotl1 | 1110032E23Rik | #N/A |  |  |
| Bptf | 2610001E17Rik | #N/A |  | B230373P09Rik | 2610001E17Rik | #N/A |  |  |
| Nsun3 | Gpd2 | #N/A |  | AK144361 | Gpd2 | #N/A |  |  |
| Gng2 | 4933430A09 | #N/A |  | Smap2 | 4933430A09 | #N/A |  |  |
| Il13ra1 | Ebf2 | #N/A |  | Paxip1 | Ebf2 | #N/A |  |  |
| Ugcg | Aps | #N/A |  | LOC378878 | Aps | #N/A |  |  |
| Ash1l | Sycp3 | #N/A |  | Clasp2 | Sycp3 | #N/A |  |  |
| G630041M05Rik | 4930429N05Rik | #N/A |  | Gpaa1 | 4930429N05Rik | #N/A |  |  |
| St3gal2 | Dbi | #N/A |  | Prdm16 | Dbi | #N/A |  |  |
| AF119384 | Lgals12 | #N/A |  | Galgt1 | Lgals12 | #N/A |  |  |
| Ccl9 | Btc | #N/A |  | Cmip | Btc | #N/A |  |  |
| Smap | Snf1lk2 | #N/A |  | 4930540E01Rik | Snf1lk2 | #N/A |  |  |
| Dck | Gpr1 | #N/A |  | 2010001J22Rik | Gpr1 | #N/A |  |  |
| Ppt1 | Folr2 | #N/A |  | Pde7b | Folr2 | #N/A |  |  |
| Ncf1 | Ybx2 | #N/A |  | Nfix | Ybx2 | #N/A |  |  |
| Sar1a | Amacr | #N/A |  | Mnt | Amacr | #N/A |  |  |
| Tfrc | Usp8 | #N/A |  | Ptbp2 | Usp8 | #N/A |  |  |
| Sri | Pcdh7 | #N/A |  | Syt13 | Pcdh7 | #N/A |  |  |
| Cdgap | Aqp7 | #N/A |  | 4432409M07Rik | Aqp7 | #N/A |  |  |
| Dlgap4 | Ntrk3 | #N/A |  | Mlc1 | Ntrk3 | #N/A |  |  |
| Ppm1a | Smoc1 | #N/A |  | 5730457F11Rik | Smoc1 | #N/A |  |  |
| Serp1 | Cacul1 | #N/A |  | Catna3 | Cacul1 | #N/A |  |  |
| Gosr1 | Pde10a | #N/A |  | Tapbp | Pde10a | #N/A |  |  |
| 2810012G03Rik | Slc25a19 | #N/A |  | Tsta3 | Slc25a19 | #N/A |  |  |
| Pycr1 | Tshr | #N/A |  | Centb1 | Tshr | #N/A |  |  |
| Sgcz | Ndufab1 | #N/A |  | B130034C11Rik | Ndufab1 | #N/A |  |  |
| Ywhae | Adrb3 | #N/A |  | Tbc1d9 | Adrb3 | #N/A |  |  |
| Prps2 | Gpd2 | #N/A |  | Zfp533 | Gpd2 | #N/A |  |  |
| Nars | AI596198 | #N/A |  | Ntn3 | AI596198 | #N/A |  |  |
| Pdpr | 2610034M16Rik | #N/A |  | Ube1c | 2610034M16Rik | #N/A |  |  |
| Nus1 | Mapbpip | #N/A |  | Kcnab2 | Mapbpip | #N/A |  |  |
| Tmed10 | AW491448 | #N/A |  | Snapc2 | AW491448 | #N/A |  |  |
| Sipa1l2 | Brp44 | #N/A |  | Nap1l5 | Brp44 | #N/A |  |  |
| Slc35a3 | Csnk | #N/A |  | Pak7 | Csnk | #N/A |  |  |
| Csnrp2 | Pkd2l2 | #N/A |  | Rpl14 | Pkd2l2 | #N/A |  |  |
| Lgals8 | Cox7a1 | #N/A |  | Rala | Cox7a1 | #N/A |  |  |
| Pum1 | Cd59a | #N/A |  | Azi2 | Cd59a | #N/A |  |  |
| Cd44 | Gpd1 | #N/A |  | Pld3 | Gpd1 | #N/A |  |  |
| 1200011I18Rik | 6530401D17Rik | #N/A |  | Ankrd29 | 6530401D17Rik | #N/A |  |  |
| Limd1 | Adipoq | #N/A |  | Aytl2 | Adipoq | #N/A |  |  |
| Irak3 | C730029A08Rik | #N/A |  | Tnrc18 | C730029A08Rik | #N/A |  |  |
| Fundc1 | Sdhc | #N/A |  | 8430438D04Rik | Sdhc | #N/A |  |  |
| 5830411E10Rik | 4930520O04Rik | #N/A |  | Ttc7 | 4930520O04Rik | #N/A |  |  |
| Stard5 | Dlat | #N/A |  | Kif21b | Dlat | #N/A |  |  |
| Ppp1cc | 2010003K11Rik | #N/A |  | Gpr132 | 2010003K11Rik | #N/A |  |  |
| Arhgap30 | Slit2 | #N/A |  | LOC224833 | Slit2 | #N/A |  |  |
| Mr1 | Pygl | #N/A |  | Arhgef1 | Pygl | #N/A |  |  |


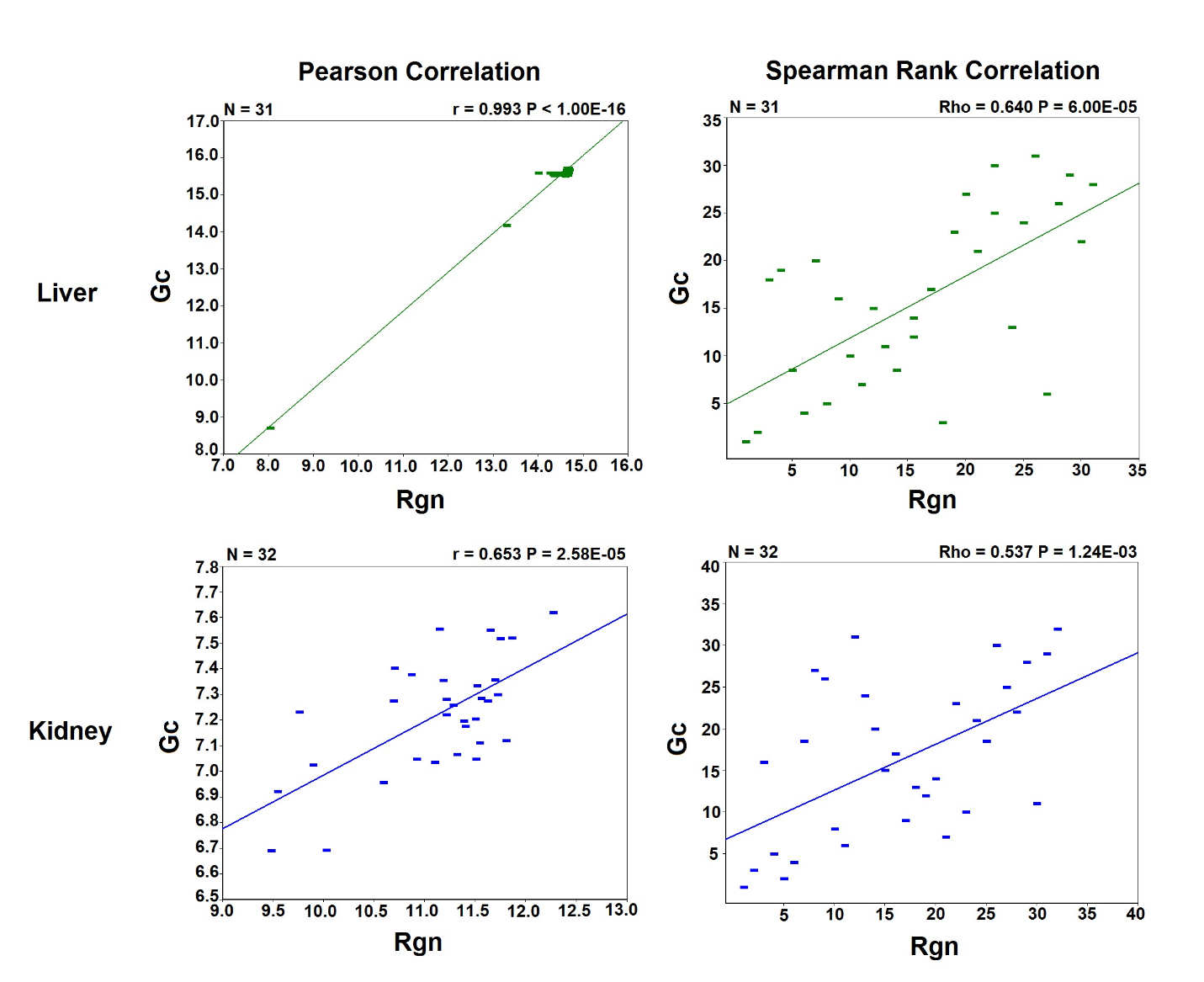


**Supplementary figure S1. The correlations in liver and kidney between Gc and Rgn in rat.** The number of samples, R values, and P values of each group are shown in the top of each graph.

**
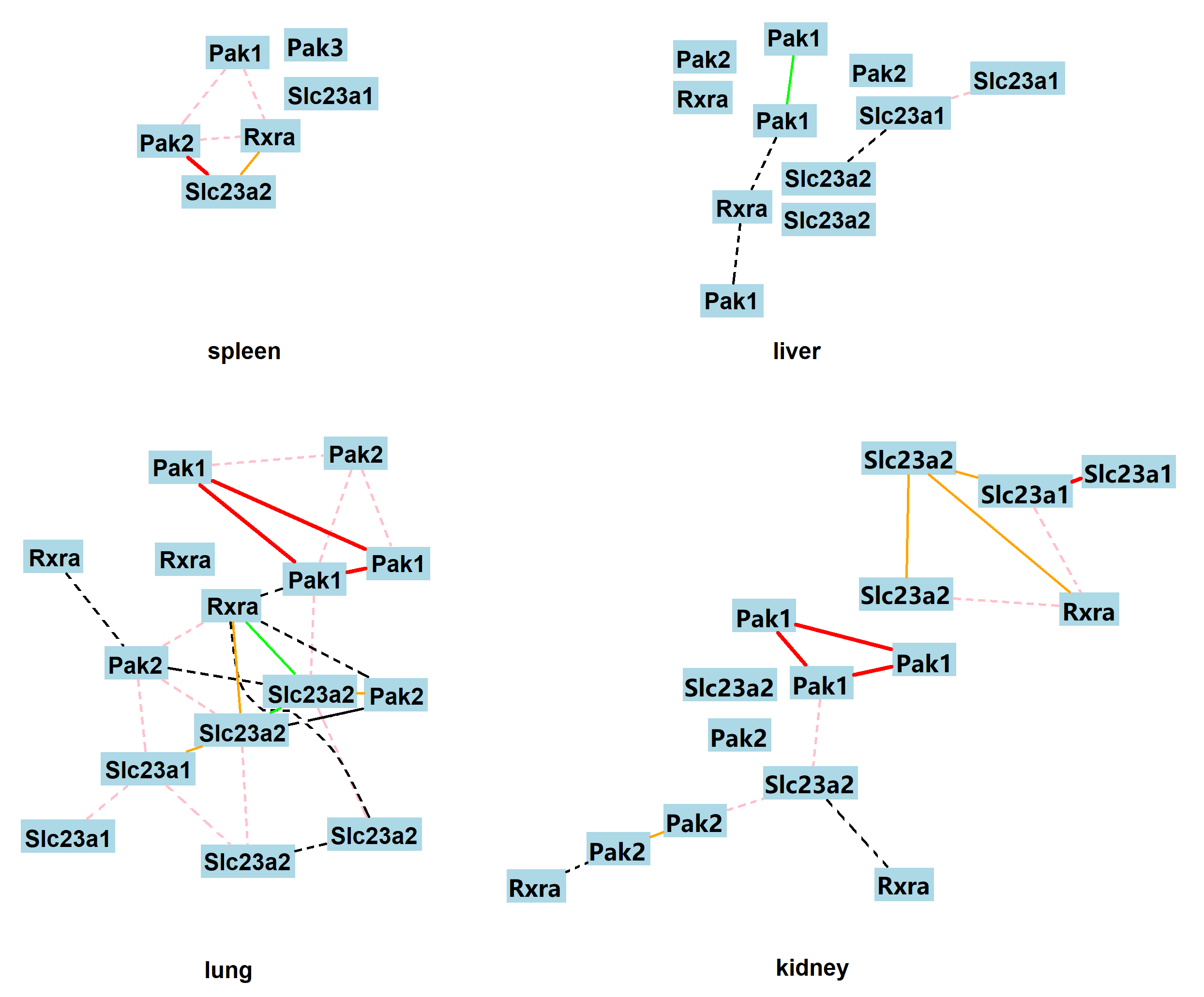
**

**Supplementary Figure S2. Correlations between PAK family and Slc23a1&2 in different tissues in mice.**

**
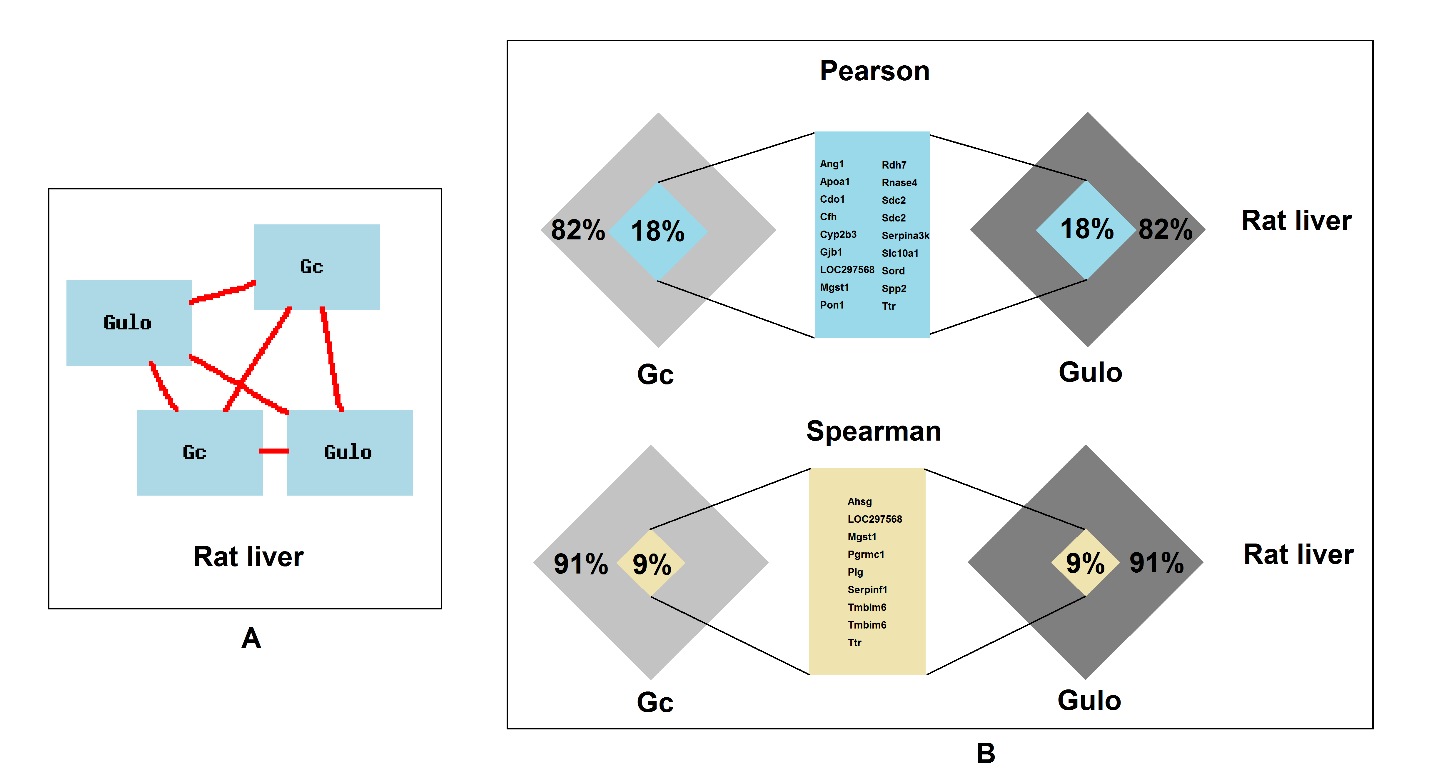
**

**Supplementary Figure S3. The rat database in GeneNetwork( MDC/CAS/UCL Liver 230v2 (Dec08) RMA Database) was used to confirm our analysis.** Each of both Gulo and GC has two probes, and they connected strongly. We chose the one with higher expression to sort the top 100 probes, as shown in figure S3. The number of same probe between Gc and Gulo in Pearson’s Rank and Spearman’ss’ Rho in rat liver is 18 and 9, respectively.
